# The Role of Cytoplasmic MEX-5/6 Polarity in Asymmetric Cell Division

**DOI:** 10.1007/s11538-021-00860-0

**Published:** 2021-02-17

**Authors:** Sungrim Seirin-Lee

**Affiliations:** Department of Mathematics, Department of Mathematical and Life Sciences, Graduate School of Integrated Science for Life, Hiroshima University, Kagamiyama 1-3-1, Hiroshima, 700-0046 Japan

**Keywords:** Pattern formation, Cell polarity

## Abstract

In the process of asymmetric cell division, the mother cell induces polarity in both the membrane and the cytosol by distributing substrates and components asymmetrically. Such polarity formation results from the harmonization of the upstream and downstream polarities between the cell membrane and the cytosol. MEX-5/6 is a well-investigated downstream cytoplasmic protein, which is deeply involved in the membrane polarity of the upstream transmembrane protein PAR in the *Caenorhabditis elegans* embryo. In contrast to the extensive exploration of membrane PAR polarity, cytoplasmic polarity is poorly understood, and the precise contribution of cytoplasmic polarity to the membrane PAR polarity remains largely unknown. In this study, we explored the interplay between the cytoplasmic MEX-5/6 polarity and the membrane PAR polarity by developing a mathematical model that integrates the dynamics of PAR and MEX-5/6 and reflects the cell geometry. Our investigations show that the downstream cytoplasmic protein MEX-5/6 plays an indispensable role in causing a robust upstream PAR polarity, and the integrated understanding of their interplay, including the effect of the cell geometry, is essential for the study of polarity formation in asymmetric cell division.

**Supplementary Information:**

The online version supplementary material available at 10.1007/s11538-021-00860-0.

## Introduction

**Fig. 1:**
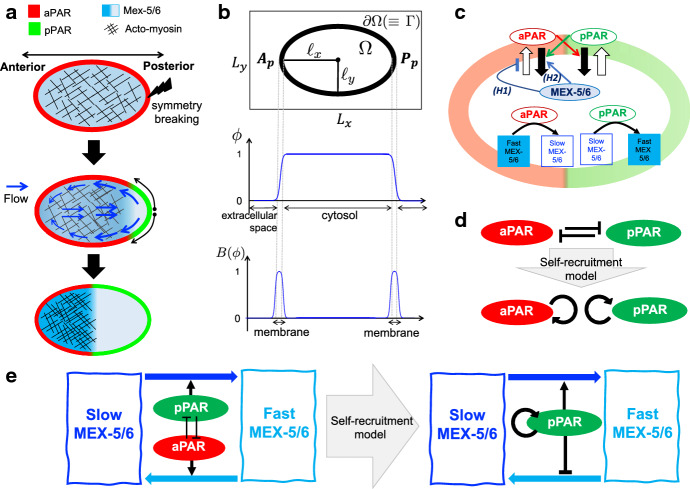
Schematic diagrams for polarity formation in the *C. elegans* embryo and mathematical model. **a** Dynamics of polarity formation in the *C. elegans* embryo. **b** The description of a cell using the phase-field function ($\phi$). $\Omega \cup \partial \Omega$ is a cell region, $L_{x}\times L_{y}$ is the simulation domain, and $\ell _{x}$ and $\ell _{y}$ are radii of the long axis and short axis of the cell, respectively. $A_p$ and $P_p$ are polar points at the anterior and posterior sides, respectively. **c** Diagram of the dynamics of aPAR, pPAR, and MEX-5/6. The black arrows indicate transportation and conversion, while the colored arrows and the inhibition symbol indicate interactions between the proteins. (H1) and (H2) indicate each assumption for how MEX-5/6 regulates aPAR. **d**, **e** Diagram of the reduction to the self-recruitment model. The black arrows and inhibition symbol indicate interaction between the proteins. The colored arrows indicate conversion of the slow and fast diffusion types of MEX-5/6

Asymmetric cell division is an elegant developmental process that creates cell diversity (Campanale et al. 2017; Knoblich 2008; Gönczy 2005). A mother cell distributes substrates and components asymmetrically before cell division and transfers them to two daughter cells, asymmetrically. Ultimately, this leads to two daughter cells with different functions and sizes. One representative experimental model of asymmetric cell division is the fertilized egg cell of *Caenorhabditis elegans* (Cuenca et al. 2002; Gönczy 2005; Motegi and Seydoux 2013). With the entry of sperm, a fertilized egg cell undergoes symmetry breaking in the posterior pole site (Fig. 1a). Concurrent with the symmetry breaking, the acto-myosin network in the cell cortex begins contracting from the site of symmetry breaking and stops contracting in the middle of the cell (Nishikawa et al. 2017; Niwayama et al. 2011). It is known that acto-myosin contraction causes cortical flow directed from the posterior to the anterior side of the cell, and cytoplasmic flow directed from the anterior to the posterior side in the center of the cell but directed from the posterior to the anterior side in the periphery of the cell membrane (Gönczy 2005; Goehring et al. 2011b; Niwayama et al. 2011) (Fig. 1a, blue arrows).

Initially, PAR-6, PAR-3, and PKC-3, a group known as anterior proteins (aPAR), are homogeneously distributed in the membrane and cytosol, while PAR-2 and PAR-1, a group known as posterior proteins (pPAR), are homogeneously distributed in the cytosol. However, once symmetry breaking occurs, these protein groups begin to form exclusive polarity domains in the membrane (Fig. 1a). pPAR relocates to the site of symmetry breaking, and aPAR relocates to the opposite site. The location of the polarity domain of these protein groups determines the anterior–posterior axis of the mother cell, and the boundary of the two exclusive polarity domains in the membrane is maintained for approximately 16 min (Gönczy 2005) after the establishment phase of the polarity, which is observed to be approximately 6–8 min (Cowan and Hyman 2004).

PAR polarity in the membrane is considered to play the central role in regulating the entire process of asymmetric cell division; therefore, both experimental and theoretical approaches to elucidate the mechanism of PAR polarity formation have been extensively studied (Cortes et al. 2018; Hoege and Hyman 2013; Lang and Munro 2017; Motegi and Seydoux 2013; Rappel and Levine 2017; Seirin-Lee 2020; Seirin-Lee et al. 2020a; Small and Dawes 2017; Zonies et al. 2010). The formation of an exclusive domain is underlined by the mutual inhibition dynamics between the anterior and posterior protein groups in which the aPAR/pPAR protein transmits pPAR/aPAR protein from the membrane to the cytosol. Theoretically, it has been demonstrated that bi-stability, due to mutual inhibition dynamics, and mass conservation are the basic mechanisms of polarity formation (Kuwamura et al. 2018; Seirin-Lee et al. 2020b; Trong et al. 2014).

Interestingly, similar polarity dynamics are also observed for cytoplasmic proteins (Cuenca et al. 2002; Daniels et al. 2010). The cytoplasmic MEX-5/6 protein simultaneously creates a polarity in the cytosol with PAR polarity formation in the membrane (Fig. 1a). The cytoplasmic MEX-5/6 protein, distributed homogeneously in the cytosol before symmetry breaking, becomes polarized to the anterior side, and the boundary of the MEX-5/6 polarity domain is observed in a location similar to the boundary of the anterior and posterior polarity domains (Cuenca et al. 2002; Schubert et al. 2000). Unlike the mechanism of PAR polarity formation in the membrane, it was found that MEX-5/6 has two different diffusive types: slow-diffusing and fast-diffusing, and it was suggested that MEX-5/6 creates polarity using the conversion dynamics of mobility. In the early stages of MEX-5/6 polarity studies, it had been hypothesized that the conversion dynamics of mobility is regulated by the phosphorylation and dephosphorylation cycle directly controlled by the membrane pPAR and aPAR proteins (Daniels et al. 2010). However, Griffin et al. (2011) suggested that pPAR (PAR-1) plays a key role and promotes the conversion from slow-diffusing MEX-5/6 to fast-diffusing MEX-5/6, but that aPAR does not play a direct role in the conversion of MEX-5/6 diffusivity. Furthermore, they hypothesized that the phosphatase PP2A antagonizes PAR-1-dependent phosphorylation of MEX-5, returning MEX-5 to the slow-diffusing state. However, aPAR (PKC-3) has been found to be significantly involved in regulating the conversion dynamics of the fast diffusive type of MEX-5/6 to the slow diffusive type (Wu et al. 2018), though it was supposed that the regulation of the conversion dynamics is likely to be indirectly regulated by aPAR proteins (Griffin et al. 2011).

While the mechanism underlying cytoplasmic polarity MEX-5/6 has been well investigated experimentally at a molecular level, a theoretical approach that integrates experimental observations is lacking. Moreover, it is not clear how MEX-5/6 polarity formation is related to the dynamics of the PARs. In particular, there has not been a study that explores how the cortical and cytoplasmic flows interact with MEX-5/6 when realistic cell geometry is included. Thus, in this study, we focus on three issues: Firstly, we formulate the MEX-5/6 model by combining it with the upstream PAR dynamics. Secondly, we explore how MEX-5/6 polarity in the cytosol affects the spatial and temporal dynamics of membrane PAR polarity. Finally, we investigate the effect of the flow dynamics and cell geometry. We explore how these two factors affect the dynamics of the cytoplasmic proteins and, consequently, the formation of membrane PAR polarity. In this study, we also introduce a general method, using phase-field modeling, to combine cell geometry with a convection–reaction–diffusion system. This method will present an easy numerical technique to simulate convection–reaction–diffusion equations on a higher-dimensional bulk-surface domain of various cell shapes.

This study suggests that it is not only the upstream polarity of PARs that dominates the downstream polarity of MEX-5/6, but also that the downstream polarity of MEX-5/6 can critically affect both the spatial and temporal dynamics of PAR polarity, and that the interaction between membrane PAR polarity and cytoplasmic MEX-5/6 polarity is vital for inducing robust cell polarity during asymmetric cell division.

## Model Development

### PARs Model

Mathematical models for the polarity formation of PAR dynamics have been proposed in several studies (Dawes and Munro 2011; Goehring et al. 2011b; Seirin-Lee and Shibata 2015; Tostevin and Howard 2008; Trong et al. 2014). All these models suggested similar mathematical structures based on bi-stability and mass conservation for the creation of polarity. Therefore, we adopt the standard model for PAR dynamics suggested by Seirin-Lee and Shibata (2015), and extend it to a higher-dimensional bulk-surface model. Let us define the cytosol by $\Omega \subset {\mathbb {R}}^{N}$ and the membrane by $\partial \Omega ~(\equiv \Gamma )$, where $\Omega$ is an open subset of ${\mathbb {R}}^{N}$ such that ${\bar{\Omega }}$ represents a cell (Fig. 1b). We also define the concentrations of anterior proteins (aPAR) in the membrane and cytosol by $[A_{m}]({\mathbf {x}},t)$ and $[A_{c}]({\mathbf {x}},t)$, respectively, and the concentrations of posterior proteins (pPAR) in the membrane and cytosol by $[P_{m}]({\mathbf {x}},t)$ and $[P_{c}]({\mathbf {x}},t)$, respectively, where ${\mathbf {x}}\in {\mathbb {R}}^{N}$ and $t\in [0, \infty )$. Then, the PAR polarity model is given by

1 $$\begin{aligned}&\dfrac{\partial [A_{m}]}{\partial t}+\nabla _{\Gamma }\cdot ({\mathbf {v}}_{m}({\mathbf {x}},t)[A_{m}]) \nonumber \\&\quad =D^{A}_{m}\nabla ^{2}_{\Gamma }[A_{m}]+F^{A}_{\text {on}}({\mathbf {x}},t)[A_{c}]-F^{A}_{\text {off}}({\mathbf {x}},t)[A_{m}] \quad \text {on~} {\mathbf {x}} \in \partial \Omega , \nonumber \\&\dfrac{\partial [A_{c}]}{\partial t}+\nabla \cdot ({\mathbf {v}}_{c}({\mathbf {x}},t)[A_{c}]) =D^{A}_{c}\nabla ^{2}[A_{c}] \quad \text {on~} {\mathbf {x}}\in \Omega , \nonumber \\&D^{A}_{c}\frac{\partial [A_{c}]}{\partial {\mathbf {n}}} -{\mathbf {v}}_{c}({\mathbf {x}},t)[A_{c}] = -F^{A}_{\text {on}}({\mathbf {x}},t)[A_{c}]+F^{A}_{\text {off}}({\mathbf {x}},t)[A_{m}] \quad \text {on~} {\mathbf {x}} \in \partial \Omega , \nonumber \\&\dfrac{\partial [P_{m}]}{\partial t}+\nabla _{\Gamma }\cdot ({\mathbf {v}}_{m}({\mathbf {x}},t)[P_{m}])\nonumber \\&\quad =D^{P}_{m}\nabla ^{2}_{\Gamma }[P_{m}]+F^{P}_{\text {on}}({\mathbf {x}},t)[P_{c}]-F^{P}_{\text {off}}({\mathbf {x}},t)[P_{m}] \quad \text {on~} {\mathbf {x}} \in \partial \Omega , \nonumber \\&\dfrac{\partial [P_{c}]}{\partial t}+\nabla \cdot ({\mathbf {v}}_{c}({\mathbf {x}},t)[P_{c}]) =D^{P}_{c}\nabla ^{2}[P_{m}] \quad \text {on~} {\mathbf {x}}\in \Omega ,\nonumber \\&D^{P}_{c}\frac{\partial [P_{c}]}{\partial {\mathbf {n}}} -{\mathbf {v}}_{c}({\mathbf {x}},t)[P_{c}] =-F^{P}_{\text {on}}({\mathbf {x}},t)[P_{c}]+F^{P}_{\text {off}}({\mathbf {x}},t)[P_{m}] \quad \text {on~} {\mathbf {x}} \in \partial \Omega , \end{aligned}$$

where ${\mathbf {n}}$ is the inner normal vector on $\partial \Omega$. Here, ${\mathbf {v}}_{m}$ and ${\mathbf {v}}_{c}$ are cortical and cytoplasmic flow velocity functions, respectively, $D_m^A$ and $D_m^P$ are the diffusion rates of aPAR and pPAR in the membrane, respectively, and $D_c^A$ and $D_c^P$ are the diffusion rates of aPAR and pPAR in the cytosol, respectively. $F_{\text {on}}^{A}$ and $F_{\text {on}}^{P}$ are the on-rate functions of aPAR and pPAR from the cytosol to the membrane, respectively, and $F_{\text {off}}^{A}$ and $F_{\text {off}}^{P}$ are the off-rate functions of aPAR and pPAR, respectively. Note that $D_c^A>D_m^A$ and $D_c^P>D_m^P$ because diffusion in the cytosol is faster than that in the membrane (Kuhn et al. 2011; Goehring et al. 2011b). We define the detailed form of the flow velocity functions in Sect. 2.5.

The off-rate functions reflect the effect of the mutual inhibition of aPAR and pPAR. aPAR/pPAR transports pPAR/aPAR from the membrane to the cytosol (Fig. 1c), and we select the functional forms suggested in Seirin-Lee and Shibata (2015):

$$\begin{aligned} F_{\text {off}}^{A}({\mathbf {x}},t)=\alpha _{1}+\frac{K_{1}[P_{m}]^{n}}{K_{2}+{\overline{K}}_2 [P_{m}]^{n}}, \qquad F_{\text {off}}^{P}({\mathbf {x}},t)=\alpha _{2}+\frac{K_{3}[A_{m}]^{n}}{K_{4}+{\overline{K}}_4[A_{m}]^{n}}, \end{aligned}$$

where $\alpha _{1}$ and $\alpha _{2}$ are basal off-rates, and $K_{1},K_{2}, {\overline{K}}_2, K_{3}, K_{4}$ and ${\overline{K}}_{4}$ are positive constants determining the off-rates. $n(>1)$ is the Hill coefficient, and we select $n=2$ for the simulations. The on-rate functions are given by

2 $$\begin{aligned} F_{\text {on}}^{A}({\mathbf {x}},t)=\gamma _{1}, \qquad F_{\text {on}}^{P}({\mathbf {x}},t)=\gamma _{2}, \end{aligned}$$

where $\gamma _1$ and $\gamma _2$ are the on-rates of aPAR and pPAR, respectively.

### MEX-5/6 Model

It is well known that MEX-5/6 has both slow and fast diffusion types in the cytosol, and that the conversion of one diffusion type to the other is regulated by PAR proteins (Daniels et al. 2010; Griffin et al. 2011; Wu et al. 2018). To develop a MEX-5/6 model combined with the PARs dynamics, we first formulate a general conversion model of MEX-5/6 diffusion. Defining the concentrations of the fast diffusive type of MEX-5/6 and slow diffusive type of MEX-5/6 by $[M_{f}]({\mathbf {x}},t)$ and $[M_{s}]({\mathbf {x}},t)$, respectively, the general MEX-5/6 conversion model is given by

3 $$\begin{aligned}&\frac{\partial [M_s]}{\partial t}+\nabla \cdot ({\mathbf {v}}_c({\mathbf {x}},t)[M_s]) \nonumber \\&\quad =D_s\nabla ^{2}[M_s]+G^C_{F\rightarrow S}({\mathbf {x}},t)[M_{f}] -G^C_{S\rightarrow F}({\mathbf {x}},t)[M_s], \quad \text {on~} {\mathbf {x}}\in \Omega , \nonumber \\&D_{s}\frac{\partial [M_{s}]}{\partial {\mathbf {n}}} -{\mathbf {v}}_{c}({\mathbf {x}},t)[M_s] \nonumber \\&\quad =G^M_{F\rightarrow S}({\mathbf {x}},t)[M_f]-G^M_{S\rightarrow F}({\mathbf {x}},t)[M_s], \quad \text {on~} {\mathbf {x}} \in \partial \Omega ,\nonumber \\&\frac{\partial [M_{f}]}{\partial t}+\nabla \cdot ({\mathbf {v}}_{c}({\mathbf {x}},t)[M_{f}]) \nonumber \\&\quad =D_{f}\nabla ^{2}[M_{f}]+G^C_{S\rightarrow F}({\mathbf {x}},t)[M_{s}]-G^C_{F\rightarrow S}({\mathbf {x}},t)[M_{f}], \quad \text {on~} {\mathbf {x}}\in \Omega , \nonumber \\&D_{f}\frac{\partial [M_{f}]}{\partial {\mathbf {n}}} -{\mathbf {v}}_{c}({\mathbf {x}},t)[M_f] \nonumber \\&\quad =G^M_{S\rightarrow F}({\mathbf {x}},t)[M_{s}]-G^M_{F\rightarrow S}({\mathbf {x}},t)[M_{f}], \quad \text {on~} {\mathbf {x}} \in \partial \Omega , \end{aligned}$$

where $D_s$ and $D_f$ are the diffusion coefficients for slow and fast diffusive types of MEX-5/6, respectively, with $D_f>D_s$. $G_{S\rightarrow F}^{\ell }({\mathbf {x}},t)$ and $G_{F\rightarrow S}^{\ell }({\mathbf {x}},t)$ are conversion functions from the slow diffusive type to the fast diffusive type, and from the fast diffusive type to the slow diffusive type, respectively, where $\ell$ denotes either cytosol (C) or membrane (M).

Next, we derive forms for $G_{S\rightarrow F}^{\ell }({\mathbf {x}},t)$ and $G_{F\rightarrow S}^{\ell }({\mathbf {x}},t)$. In the wild type of *C. elegans*, the diffusion rate of MEX-5/6 in the posterior side is notably higher than in the anterior side. The studies by Daniels et al. (2010) and Griffin et al. (2011) suggest that pPAR (PAR-1) regulates the slow type of MEX-5/6, and the diffusion rate of MEX-5/6 of the posterior side in the PAR-1 mutant cell is significantly decreased compared to the wild type. This result indicates that PAR-1 promotes the conversion dynamics of the slow type of MEX-5/6 to the fast type. Thus, we suppose that the conversion rate from the slow type to the fast type has a positive correlation with pPAR concentration, and we propose that

$$\begin{aligned} G^M_{S\rightarrow F}({\mathbf {x}},t)=\mu _1 [P_m]({\mathbf {x}},t), \quad G^C_{S\rightarrow F}({\mathbf {x}},t)=\mu _3 [P_c]({\mathbf {x}},t), \end{aligned}$$

where $\mu _1$ and $\mu _3$ are positive correlation constants reflecting the effective strength of pPAR on the conversion rate around the cell membrane and within the bulk cytosol, respectively.

The study by Wu et al. (2018) suggests that the diffusion rate of MEX-5/6 in the anterior side of the aPAR(PKC-3) mutant cell is significantly increased compared to the wild type. This result suggests two hypotheses: either PKC-3 promotes the conversion dynamics of the fast type of MEX-5/6 to the slow type, or PKC-3 plays a role in inhibiting the conversion dynamics from the slow type to the fast type of MEX-5/6. However, the diffusion rates of both PKC-3 and PAR-1 mutant cells did not show a notable difference from that of only PKC-3 mutant cell. This indicates that PKC-3 does not play the latter role, and it may promote the conversion of fast type to slow type of MEX-5/6. Thus, we suppose that the conversion rate from the fast type to the slow type has a positive correlation with aPAR concentration, and this leads us to define

4 $$\begin{aligned} G^M_{F\rightarrow S}({\mathbf {x}},t)=\mu _2 [A_m]({\mathbf {x}},t), \quad G^C_{F\rightarrow S}({\mathbf {x}},t)=\mu _4 [A_c]({\mathbf {x}},t), \end{aligned}$$

where $\mu _2$ and $\mu _4$ are positive correlation constants reflecting the effective strength of aPAR on the conversion rate in the cell membrane and bulk cytosol, respectively.

### MEX-5/6-Combined-PARs Model

Experimental observations of the *C. elegans* embryo suggest that MEX-5/6 regulates the expansion of the pPAR domain by helping to exclude the aPAR domain, rather than directly promoting pPAR localization (Cuenca et al. 2002; Schubert et al. 2000), which suggests the possibility that MEX-5/6 may directly regulate the translocation dynamics of aPAR between the membrane and the cytosol. On the other hand, the detailed molecular mechanism of the interaction between MEX-5/6 and the PARs is still unclear. Thus, we consider two possible assumptions. We suppose that the experimental observation of Cuenca et al. (2002) is related to either the on-rate or the off-rate of aPAR in the model (1). Thus, we assume that in one model, MEX-5/6 inhibits the recruitment of aPAR from the cytosol to the membrane (i.e., the on-rate of aPAR), and in the other model, we assume that MEX-5/6 promotes the transport of aPAR from the membrane to the cytosol (i.e., the off-rate of aPAR). We call these models *H1* and *H2*, respectively (Fig. 1c). We formulate the simplest type of model as follows:

5 $$\begin{aligned} \begin{aligned}&\textit{H1}: \qquad F_{\text {on}}^{A,H_1}({\mathbf {x}},t)=\frac{F_{\text {on}}^{A}({\mathbf {x}},t)}{1+\mu _0[M]({\mathbf {x}},t)}, \\&\textit{H2}: \qquad F_{\text {off}}^{A,H_2}({\mathbf {x}},t)=F_{\text {off}}^{A}({\mathbf {x}},t)(1+\mu _0[M]({\mathbf {x}},t)), \end{aligned} \end{aligned}$$

where $[M]({\mathbf {x}},t)=[M_{f}]({\mathbf {x}},t)+[M_{s}]({\mathbf {x}},t)$, and $\mu _{0}$ is either the inhibition rate of aPAR recruitment or the promotion rate of aPAR transport from the membrane to cytosol by MEX-5/6. Note that $\mu _0=0$ recovers the original model (1) without the effect of MEX-5/6. We name the combination of model (1) including (5), with model (3), the *MEX-5/6-combined-PARs Model* (Fig. 1c).

### MEX-5/6-Combined-Self-Recruitment pPAR Model

The self-recruitment model of PAR dynamics was first suggested in Seirin-Lee and Shibata (2015), in which the aPAR-pPAR model is reduced to either an aPAR alone or a pPAR alone model, and the effect of the off-rate (by the mutual inhibition) is replaced by a self-recruitment effect resulting from either aPAR or pPAR itself. By applying this reduction to the self-recruitment model, we can study the interaction of PAR polarity dynamics with pPAR alone. This gives us more precise information on how pPAR is directly, or indirectly, involved in MEX-5/6 dynamics (Fig. 1d, e). Thus, we here reduce the MEX-5/6-combined-PARs Model (1)–(3) to the self-recruitment form and show that the conversion model of MEX-5/6 by aPAR and pPAR given in (3) is essentially equivalent to the conversion model through pPAR alone.

The polarity of the PARs is formed only in the membrane. We find that the interface between the aPAR and pPAR domains in the membrane is sufficiently narrow and that the concentration of pPAR in the membrane is very low where the concentration of aPAR is high (Fig. 3a, middle panel). Thus, we approximate the effect of the off-rate of aPAR ($F_{\text {off}}^A$) under the condition that $[P_{m}]\ll 1$ by Taylor expansion :

$$\begin{aligned} F_{\text {off}}^{A}({\mathbf {x}},t)=\alpha _{1}+\frac{K_{1}[P_{m}]^{n}}{K_{2}+{\overline{K}}_2 [P_{m}]^{n}}\approx \alpha _{1}+F'(0)[P_{m}]+\frac{F''(0)}{2}[P_{m}]^2+O([P_{m}]^3). \end{aligned}$$

We can easily calculate $F'(0)=0$ for $n\ge 2$, $F''(0)=2K_1K_2^{-1}$ for $n=2$ and $F''(0)=0$ for $n\ge 3$. Assuming $n=2$, we obtain

6 $$\begin{aligned} F_{\text {off}}^{A}({\mathbf {x}},t)\approx \alpha _{1}+\frac{K_1}{K_2}[P_{m}]^2+O([P_{m}]^3). \end{aligned}$$

We assume that the fast diffusion of aPAR in the cytosol leads to a well-mixed state and that the concentration of aPAR in the cytosol quickly approaches an equilibrium state, namely $A_c^*=(1/\Omega )\int _{\Omega }[A_c]({\mathbf {x}},t)d{\mathbf {x}}$. This leads to

$$\begin{aligned} -F^{A, H_1}_{\text {on}}({\mathbf {x}},t)A_c^*+F^{A}_{\text {off}}({\mathbf {x}},t)[A_{m}] \approx 0 ~~\text {for \textit{H1}},\\ -F^{A}_{\text {on}}({\mathbf {x}},t)A_c^*+F^{A, H_2}_{\text {off}}({\mathbf {x}},t)[A_{m}] \approx 0 ~~\text {for \textit{H2}}. \end{aligned}$$

With the approximation (6) and the on-rate function $F^{A}_{\text {on}}$ (2), we have the same approximation for both *H1* and *H2*, such that

7 $$\begin{aligned}{}[A_m]\approx \frac{F_{\text {on}}^{A}({\mathbf {x}},t)A_c^*}{(1+\mu _0[M])F_{\text {off}}^{A}({\mathbf {x}},t)}=\frac{\delta _2}{(1+\mu _0[M])(1+\delta _1[P_m]^2)} \end{aligned}$$

where $\delta _1=K_1/(K_2\alpha _1)$ and $\delta _2=\gamma _1 A_c^*/\alpha _1$. Thus, both the *H1* and *H2* models are essentially the same, and the effect of MEX-5/6 on aPAR dynamics is likely to decrease the concentration of aPAR in the membrane.

Substituting the approximate equation (7) for $[A_m]$ into the off-rate function $F_{\text {off}}^P$ of pPAR, (2), we obtain

8 $$\begin{aligned} F_{\text {off}}^{P}({\mathbf {x}},t)=\alpha _2+\frac{\beta _4}{\beta _1+(1+\mu _0[M])^2(\beta _2+\beta _3[P_m]^2)+O([P_m]^4)}, \end{aligned}$$

where $\beta _1=\overline{K}_4 \delta _2^2, ~\beta _2=K_4, ~\beta _3=2K_4 \delta _1$, and $\beta _4=K_3 \delta _2^2$. Finally, $G^C_{F\rightarrow S}({\mathbf {x}},t)$ and $G^M_{F\rightarrow S}({\mathbf {x}},t)$, given in (4), are transformed to

9 $$\begin{aligned} G^C_{F\rightarrow S}({\mathbf {x}},t)=\mu _4 A_c^*, \quad G^M_{F\rightarrow S}({\mathbf {x}},t)=\mu _2 \frac{\delta _2}{(1+\mu _0[M])(1+\delta _1[P_m]^2)} \end{aligned}$$

from equation (7).

Combining Eqs. (6) and (9) with the pPAR equations of the model (1) and the MEX-5/6 model (3), we obtain the *MEX-5/6-combined-self-recruitment pPAR Model* as follows:

10 $$\begin{aligned}&\frac{\partial [P_{m}]}{\partial t}+\nabla _{\Gamma }\cdot ({\mathbf {v}}_{m}[P_{m}])=D^{P}_{m}\nabla ^{2}_{\Gamma }[P_{m}]\nonumber \\&\quad +\gamma _2[P_{c}]-\left\{ \alpha _2+\frac{\beta _4}{\beta _1+(1+\mu _0[M])^2(\beta _2+\beta _3[P_m]^2)} \right\} [P_{m}], \text {on~} {\mathbf {x}} \in \partial \Omega , \nonumber \\&\frac{\partial [P_{c}]}{\partial t}+\nabla \cdot ({\mathbf {v}}_{c}[P_{c}]) =D^{P}_{c}\nabla ^{2}[P_{m}], \text {on~} {\mathbf {x}}\in \Omega ,\nonumber \\&D^{P}_{c}\frac{\partial [P_{c}]}{\partial {\mathbf {n}}} -{\mathbf {v}}_{c}({\mathbf {x}},t)[P_{c}]\nonumber \\&\quad =-\gamma _2[P_{c}]+\left\{ \alpha _2+\frac{\beta _4}{\beta _1+(1+\mu _0[M])^2(\beta _2+\beta _3[P_m]^2)} \right\} [P_{m}], \text {on~} {\mathbf {x}} \in \partial \Omega ,\nonumber \\ \end{aligned}$$

and

11 $$\begin{aligned}&\dfrac{\partial [M_{s}]}{\partial t}+\nabla \cdot ({\mathbf {v}}_{c}[M_{s}]) \nonumber \\&\quad =D_{s}\nabla ^{2}[M_{s}] +\mu _4 \delta _3[M_{f}]-\mu _3 [P_c][M_{s}], \quad \text {on~} {\mathbf {x}}\in \Omega , \nonumber \\&D_{s}\dfrac{\partial [M_{s}]}{\partial {\mathbf {n}}} -{\mathbf {v}}_{c}[M_s]\nonumber \\&\quad =\mu _2 \frac{\delta _2}{(1+\mu _0[M])(1+\delta _1[P_m]^2)}[M_f]-\mu _1 [P_m][M_s], \quad \text {on~} {\mathbf {x}} \in \partial \Omega ,\nonumber \\&\dfrac{\partial [M_{f}]}{\partial t}+\nabla \cdot ({\mathbf {v}}_{c}[M_{f}])\nonumber \\&\quad =D_{f}\nabla ^{2}[M_{f}]+\mu _3 [P_c][M_{s}]-\mu _4 \delta _3[M_f], \quad \text {on~} {\mathbf {x}}\in \Omega ,\nonumber \\&D_{f}\dfrac{\partial [M_{f}]}{\partial {\mathbf {n}}} -{\mathbf {v}}_{c}[M_f]\nonumber \\&\quad =\mu _1 [P_m][M_s]-\mu _2 \frac{\delta _2}{(1+\mu _0[M])(1+\delta _1[P_m]^2)}[M_f], \quad \text {on~} {\mathbf {x}} \in \partial \Omega , \end{aligned}$$

where we have replaced $A_c^*$ by a positive parameter, $\delta _3$, without loss of generality. The model is a conservation system and the total mass of pPAR and MEX-5/6 is conserved.

From a direct comparison between the MEX-5/6-combined-PARs Model (1)–(3) and MEX-5/6-combined-self-recruitment pPAR Model (10)–(11), we find that the regulation network involving aPAR, pPAR, and MEX-5/6 can be reduced to the network of pPAR-alone conversion control in the MEX-5/6 dynamics (Fig. 1e). We can interpret the model (11) such that conversion from the fast diffusive type to slow diffusive type of MEX-5/6 is promoted constantly (the terms $\mu _4\delta _3$ and $\mu _2\delta _2$) by a substrate and it is simultaneously down-regulated by pPAR in the membrane. The substrate may be considered to be phosphatase PP2A, and the inhibition by pPAR on the membrane may be considered as an indirect role of aPAR on the conversion of MEX-5/6, as suggested in Griffin et al. (2011) and Wu et al. (2018). Our model reduction suggests that the direct conversion model involving aPAR and pPAR is essentially the same as the pPAR-alone conversion model. Indeed, we confirm that the two models essentially show similar dynamics (see Fig. 3). In this paper, we explore our results on the MEX-5/6-combined-self-recruitment pPAR Model (10)–(11).

**Fig. 2:**
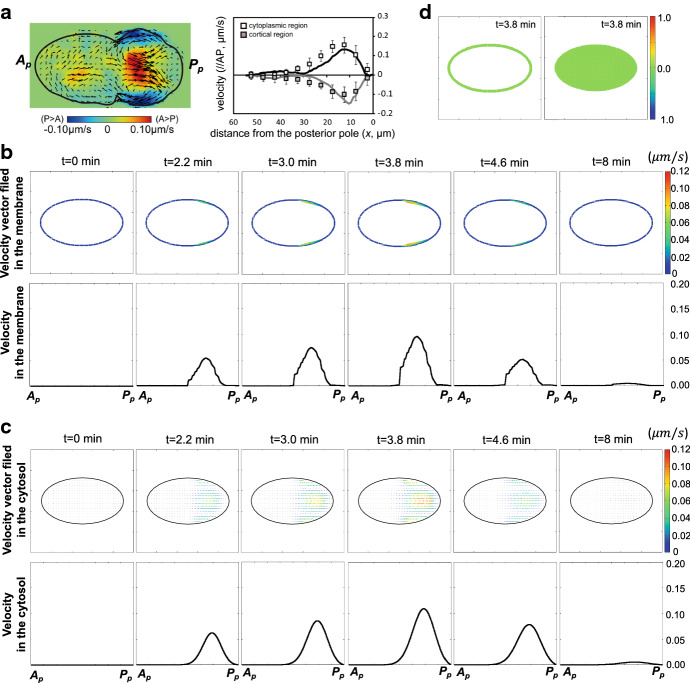
Flow velocity function. **a** Experimental data of flow velocity (adapted from Niwayama et al. (2011)). Right panel shows the velocity distribution of cytoplasmic streaming. Lines in the left panel show the velocity distribution along the AP axis in vivo reconstructed with the moving particle simulation. Boxes in the left panel show the experimental data (gray: cortical flow, white: cytoplasmic flow). **b**, **c** Representative example of the flow velocity function ${\mathbf {v}}({\mathbf {x}}, t)=(v^x, v^y)$ given by (12). The vectors indicate the direction, and the color map shows the speed of flow velocities. The one-dimensional velocity data ($|{\mathbf {v}}|$) in the membrane have been plotted on the cell circumference, the data in the cytosol have been plotted on $(x, y)=(x, L_y/2)$. **d** A representative simulation of incompressibility at maximal flow velocity. The numerical simulations show that $\nabla \cdot {\mathbf {v}}\approx O(2\times 10^{-2})$ during the flow

### Flow Velocity Function

After sperm entry, the acto-myosin network located in the cell cortex begins contracting toward the anterior side from the posterior side (Gönczy 2005) (Fig. 1a), causing a cortical flow in the same direction as the acto-myosin contraction, and a cytoplasmic flow in the opposite direction of the center of the cytosol and in the same direction as the cortical flow in the periphery of the membrane (Niwayama et al. 2011) (Fig. 2a). The velocity of the flows has been investigated in detail experimentally, revealing that the maximal velocity (approximately 0.156±0.044 $\mu m/s$) is reached during the establishment phase and the velocity goes to zero around the time that the establishment phase terminates (Goehring et al. 2011b; Niwayama et al. 2011). In this study, we explicitly formulate the flow velocity function, ${\mathbf {v}}({\mathbf {x}},t)=(v^{x}({\mathbf {x}},t), v^{y}({\mathbf {x}},t))$, defined for the entire cell region (${\bar{\Omega }}$) based on the experimental data in Niwayama et al. (2011).

The flow velocity function is given by

12 $$\begin{aligned} \begin{aligned}&v^{x}({\mathbf {x}},t)=\frac{c_{3}[x-(L_{x}/2-\ell _{x})]^2[(L_{x}/2+\ell _{x})-x]\cos (c_{4}\pi (y-L_{y}/2))}{c_{1}+\exp [c_{2}(x-L_{x})^2/(1+c_{5}t)]}\times \tau (t),\\&v^{y}({\mathbf {x}},t)= \frac{ c_8 [x-(L_{x}/2+\ell _{x}/2)]\sin {(c_7 \pi (y-L_y/2))}}{c_{1}+\exp [c_{2}(x-(L_{x}/2+\ell _{x}/2))^2/(1+c_{5}t)]} \times \tau (t), \end{aligned} \end{aligned}$$

where

$$\begin{aligned} \tau (t)=\left\{ \begin{array}{cc}\frac{t}{T_0}\{c_6(T_0-t)+1\} &{} t<T_0 \\ \frac{1}{1+c_9(t-T_0)^2}&{} t\ge T_0\end{array}\right. \end{aligned}$$

and $c_i(i=1\ldots 9)$ are positive constants. $T_{0}$ is the temporal point at which the velocity is maximum. We select the parameter values so that the flow velocity function approaches the maximal velocity of about 0.12 $\upmu$ m/s at 3.8 min, and the flow ceases around 8 min (Fig. 2b, c, Movie S1). The cortical flow velocity function, ${\mathbf {v}}_{m}({\mathbf {x}},t)$, is given by the value of $(v^{x}({\mathbf {x}},t), v^{y}({\mathbf {x}},t))$ on the domain $\partial \Omega$, and the cytoplasmic flow velocity function, ${\mathbf {v}}_{c}({\mathbf {x}},t)$, is given by the value of $(v^{x}({\mathbf {x}},t), v^{y}({\mathbf {x}},t))$ on the domain $\Omega$. Detailed temporal and spatial data for the cortical and cytoplasmic flows used in our simulations are shown in Fig. 2b, c. Note that the flow functions in a cell satisfy incompressibility almost everywhere (Fig. 2d and Fig. S1)

### Model Incorporated with the Bulk-Surface Cellular Geometry

Here, we introduce a method to combine the phase-field function with the bulk-surface system (10)–(11). The method allows for a simple numerical technique to solve a convection–reaction–diffusion model on any high-dimensional cellular shape, and we can also simply include the flow dynamics in the model system. Let us express a fixed cell domain using a phase-field function for some time $t^*$, namely $\phi ({\mathbf {x}},t^*)$, as follows (Fig. 1b):

$$\begin{aligned}&\text {Cytosol}\equiv \{{\mathbf {x}}|\phi ({\mathbf {x}},t^*)=1 \}, \quad \text {Membrane}\equiv \{{\mathbf {x}}|0<\phi ({\mathbf {x}},t^*)<1\}, \\&\text {Extracellular region}\equiv \{{\mathbf {x}}|\phi ({\mathbf {x}},t^*)=0\}. \end{aligned}$$

We now explain a general method to make a phase-field function of a cell. Let us first define the free energy function, $E_0$, of Ginzburg–Landau type for a cell such that

$$\begin{aligned} E_{0}&=\int _{A} \frac{\varepsilon ^2}{2}| \nabla \phi |^2+g(\phi )\mathrm{d}x \end{aligned}$$

where *A* denotes the area of the system in which $\phi$ is defined, $\varepsilon (>0)$ is a sufficiently small constant that defines the thickness of the cell membrane, and $g(\phi )=\frac{1}{4}\phi ^2(1-\phi )^2$. Here, the symmetric potential $g(\phi )$ is used for setting the local minima at $\phi =0$ and $\phi =1$.

Next, we define the energy function which determines the volume of the cell such that

$$\begin{aligned} E_{1}&=\alpha \left( \int _{A}h(\phi )\mathrm{d}{{\mathbf {x}}}-\overline{V}\right) ^2, \end{aligned}$$

where $\alpha (>0)$ is the intensity constant of the energy for cell volume, $\overline{V}$ is the target volume of the cell, and $h(\phi )=\phi ^3(10-15\phi +6\phi ^2)$, which is used for the induction of an energetic asymmetry between $\phi =0$ and $\phi =1$, driving the interface while keeping $\phi =0$ and $\phi =1$ as local minima of the energy function (see Appendix of Seirin-Lee et al. (2017) for more detail). Finally, we define a time evolution equation for the total energy of the cell, satisfying

$$\begin{aligned} \mu ^{-1}\frac{\partial \phi }{\partial t}=-\frac{\delta (E_{0}+E_{1})}{\delta \phi }, \end{aligned}$$

where $\mu (>0)$ is the constant defining the mobility of the interface. Substituting for $E_0$ and $E_1$ into the above equation, we arrive at the equation

13 $$\begin{aligned} \mu ^{-1}\frac{\partial \phi }{\partial t}=\varepsilon ^2\nabla ^2\phi +\phi (1-\phi )\left\{ \phi -\frac{1}{2}-60\alpha \phi (1-\phi )\left( V(t)-\overline{V}\right) \right\} . \end{aligned}$$

By providing the target cell volume (${\bar{V}}$) and initial conditions, we can readily generate cells that have different shapes and sizes. We generate the *C. elegans* embryo by setting $\overline{V}$ as the actual size (the area in two-dimensional simulations) of the embryo and the initial condition to be an ellipse with the embryo scale of short and long axes.

With the cell phase-field function $\phi$, we rewrite the MEX-5/6-combined-self-recruitment pPAR model (10)–(11) in a form in which the cell geometry is reflected (Seirin-Lee 2016; Teigen et al. 2009; Wang et al. 2017). The model system, combined with the phase-field function that we used for numerical simulations, is given by

14 $$\begin{aligned}&\frac{\partial B(\phi )[P_{m}]}{\partial t}+\nabla \cdot (B(\phi ){\mathbf {v}}_{m}[P_{m}] )= D^{P}_{m}\nabla \cdot (B(\phi )\nabla [P_{m}])\nonumber \\&\quad +B(\phi )\left[ \gamma _2[P_{c}]-\left\{ \alpha _2+\frac{\beta _4}{\beta _1+(1+\mu _0[M])^2(\beta _2+\beta _3[P_m]^2)} \right\} [P_{m}]\right] , \nonumber \\&\frac{\partial \phi [P_{c}]}{\partial t} +\nabla \cdot (\phi {\mathbf {v}}_{c}[P_{c}] )= D^{P}_{c}\nabla \cdot (\phi \nabla [P_{c}])\nonumber \\&\quad +|\nabla \phi |\left[ -\gamma _2[P_{c}]+\left\{ \alpha _2+\frac{\beta _4}{\beta _1+(1+\mu _0[M])^2(\beta _2+\beta _3[P_m]^2)} \right\} [P_{m}]\right] , \nonumber \\&\frac{\partial \phi [M_{s}]}{\partial t} +\nabla \cdot (\phi {\mathbf {v}}_{c}[M_{s}]) = D_{s}\nabla \cdot (\phi \nabla [M_{s}])+\phi \{\mu _4 \delta _3[M_{f}]-\mu _3 [P_c][M_{s}]\}\nonumber \\&\quad +|\nabla \phi | \left\{ \mu _2 \frac{\delta _2}{(1+\mu _0[M])(1+\delta _1[P_m]^2)}[M_{f}]-\mu _1 [P_m][M_{s}] \right\} ,\nonumber \\&\frac{\partial \phi [M_{f}]}{\partial t} +\nabla \cdot (\phi {\mathbf {v}}_{c}[M_{f}]) = D_{f}\nabla \cdot (\phi \nabla [M_{f}])+\phi \{\mu _3 [P_c][M_{s}]-\mu _4 \delta _3[M_{f}]\}\nonumber \\&\quad +|\nabla \phi | \left\{ \mu _1 [P_m][M_{s}]-\mu _2 \frac{\delta _2}{(1+\mu _0[M])(1+\delta _1[P_m]^2)}[M_{f}] \right\} , \end{aligned}$$

for ${\mathbf {x}}\in A(\equiv [0, L_x]\times [0, L_y])$, where $B(\phi )=\nu \phi ^{2}(1-\phi )^2~(\nu >0)$, a function defining the membrane region (Fig. 1b). The cortical flow velocity function, ${\mathbf {v}}_{m}({\mathbf {x}},t)$, is given by ${\mathbf {v}}_{m}({\mathbf {x}},t)=B(\phi ({\mathbf {x}},t)){\mathbf {v}}({\mathbf {x}},t)$, and the cytoplasmic flow velocity function, ${\mathbf {v}}_{c}({\mathbf {x}},t)$, is given by ${\mathbf {v}}_{c}({\mathbf {x}},t)=\phi ({\mathbf {x}},t){\mathbf {v}}({\mathbf {x}},t)$.

One can confirm that a sharp interface limit recovers the boundary conditions in the cytosol equations of the phase-field combined model (see “Appendix A” for more detail). Note that we can numerically solve the bulk-surface model (10)–(11) using a standard finite difference method on a square. The details of the initial conditions and parameter values are given in “Appendix B.”

**Fig. 3:**
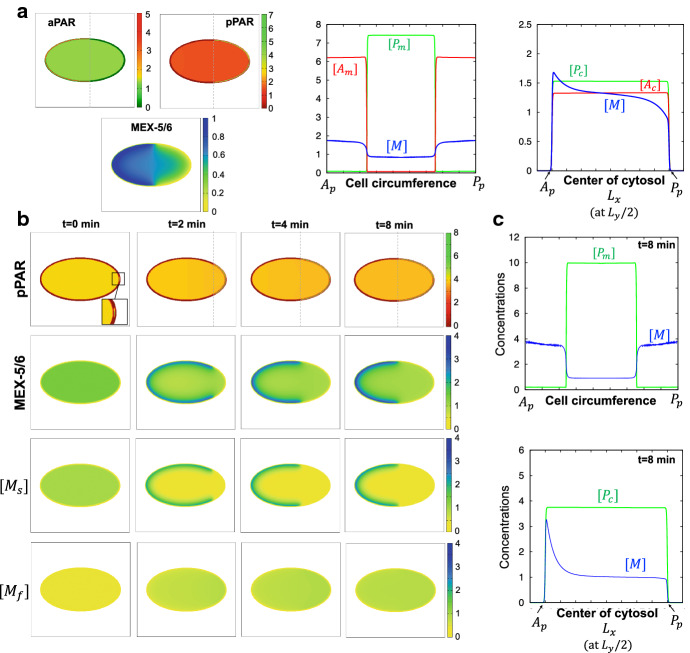
Dynamics of PARs and MEX-5/6 polarity formation. **a** Representative simulation results of the MEX-5/6-combined-PAR model (1)–(3) without flow. Left panels show the polarities of aPAR, pPAR, and MEX-5/6. The concentrations in the cell circumference are plotted in the middle panel and the concentrations in the cytosol are plotted in the right panel. **b**, **c** Representative simulation results of the MEX-5/6-combined-self-recruitment pPAR model (10)–(11) without flow. The gray dotted line indicates the boundary location of the polarity domains. The detailed parameter values are given in “Appendix B”

**Fig. 4:**
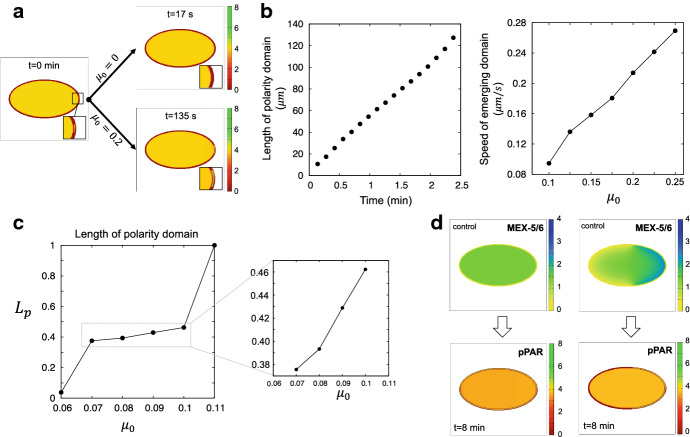
Role of MEX-5/6 polarity on PAR polarity formation. **a** The effect of MEX-5/6 on the symmetry breaking phase. **b** The effect of MEX-5/6 on the establishment phase. The left panel shows the change in length of the pPAR domain and the right panel shows the effect of MEX-5/6 on the emerging speed of the pPAR polarity domain. The data were measured using the average speed over the interval [0.5 min, 1 min] for each simulation. **c** The effect of MEX-5/6 on the maintenance phase. The effect of MEX-5/6 on the length of the pPAR polarity domain is shown. **d** The effect of MEX-5/6 polarity on pPAR polarity. The upper panels show how the polarity of MEX-5/6 was numerically controlled and the lower panels show the resultant pPAR polarity for each case. The detailed parameter values are given in “Appendix B”

## Results

### Regeneration of PAR and MEX-5/6 Polarities

We first confirm that the MEX-5/6-combined-PARs model (1)–(3) and the MEX-5/6-combined-self-recruitment pPAR model (10)–(11) are essentially the same (Fig. 3), and there are no qualitative differences in the model dynamics, suggesting that the two different conversion dynamics suggested by Daniels et al. (2010) and Griffin et al. (2011) can be reconsidered by our mathematical models, and they have essentially the same mathematical structure. This implies that our model is integrating all relevant molecular dynamics observed in the previous experiments and is thus a general model to capture the dynamics of both MEX-5/6 and PAR, simultaneously.

In our model, we confirmed that PAR polarity in the membrane and MEX-5/6 polarity in the cytosol are simultaneously generated (Fig. 3b, Movie S2, S3), as observed experimentally (Cuenca et al. 2002). The simulations showed that the establishment phase finishes at approximately 6–7 min, and the boundary of the pPAR domain stops at around the middle of the cell, for a representative parameter set. With a small initial stimulus at the posterior polar site (Fig. 3b, $t=0$ panel), the pPAR domain begins to emerge toward the anterior from the posterior, and simultaneously MEX-5/6 generates polarity in the same direction on the emergence of pPAR. The domain boundary of MEX-5/6 is always determined in a location similar to the domain boundary of pPAR in the periphery of the membrane (Fig. 3c, upper panel). This result suggests that polarity formation of MEX-5/6 and PAR is very interactive, both temporally and spatially.

On the other hand, the MEX-5/6 concentration profile in the bulk region of the cytosol shows that the distribution of MEX-5/6 is not clearly distinguished by the two domains of different concentration levels (Fig. 3c, lower panel). This is likely to be a consequence of the homogeneity of the pPAR concentration in the cytosol (Seirin-Lee et al. 2020b). We further found that the slow diffusive MEX-5/6 has a similar distribution shape to that of the total MEX-5/6, whereas the fast diffusive MEX-5/6 is almost homogeneous. This indicates that the conversion of MEX-5/6 to a slow diffusive type is essential to create the MEX-5/6 polarity, and that the inhibition/activation role of pPAR on the conversion of MEX-5/6 to a slow/fast diffusive type is critical. Our bulk-surface model proposes that the polarity of MEX-5/6 is mainly formed in the periphery of the cell membrane rather than in the bulk space of the cytosol, and that the heterogeneity of pPAR polarity in the membrane plays an important role in generating MEX-5/6 polarity.

### Role of MEX-5/6 Polarity on PAR Polarity Formation

How the upstream PAR proteins, and their polarity, influence formation of MEX-5/6 have been investigated in detail, both experimentally and mathematically (Wu et al. 2018; Seirin-Lee et al. 2020b). However, it is still unknown how the downstream MEX-5/6 polarity influences the upstream PAR polarity. Thus, we explore here the biochemical roles of MEX-5/6 on PAR polarity formation by investigating how MEX-5/6 affects PAR polarity formation, spatially and temporally, with respect to the symmetry breaking, establishment, and maintenance phases, without flow effects. To see the influence on the symmetry breaking phase, we investigated whether the polarity pattern can emerge for the cases when the effect of MEX-5/6 is absent ($\mu _0=0$) or present ($\mu _0>0$). We found that MEX-5/6 can promote symmetry breaking (Fig. 4a), implying that MEX-5/6 supports pPAR invasion into the membrane by suppressing aPAR. To see how MEX-5/6 controls pPAR recruitment, we analyzed how the property of the bi-stability of pPAR dynamics can be affected by the parameter $\mu _0$ (see “Appendix D”). The analysis showed that $\mu _0$ leads to a wider parameter region for the bi-stability of pPAR dynamics (Fig. S2), suggesting that MEX-5/6 plays an important supporting role in the formation of pPAR polarity.

Next, we investigated the emerging speed of polarity pattern in the establishment phase (Fig. 4b). We found that the speed is almost constant (Fig. 4b, left panel) before it enters the maintenance phase, in which the speed should be slower in order to halt the speed of establishment polarity pattern. Thus, we explored how the emerging speed is affected by MEX-5/6, namely $\mu _0$, during the early stage of the establishment phase (Fig. 4b, right panel). The results show that the emerging speed is highly affected by changes in $\mu _0$. The emerging speed increased by more than double its value when $\mu _0$ doubled in magnitude, indicating that MEX-5/6 plays a critical role in regulating the temporal dynamics of PAR polarity formation.

Finally, to see the effect of MEX-5/6 in the maintenance phase, we focused on two phenomena: one is the length scale of the pPAR polarity domain ($L_p=$ [Length of polarity domain]/[Length of cell circumference]) and the other is the location of pPAR polarity. We first found that the length scale of the pPAR domain can be affected by MEX-5/6 (Fig. 4c). As $\mu _0$ increased, the length of the pPAR domain increased. The total mass of proteins is conserved; therefore, it is likely that the MEX-5/6 redistributed the pPAR protein between the membrane and cytosol through helping pPAR stay in the membrane. However, the parameter range of $\mu _0$ where pPAR forms a stationary polarity pattern was approximately within 8% variation of the length scale, and there existed two threshold values of $\mu _0$ at which pPAR either fails to invade, or spreads throughout the whole cell membrane. This indicates that the effect of MEX-5/6 on the length scale of the pPAR domain may be negligible, but the maintenance of pPAR polarity may be tightly regulated by MEX-5/6.

We also found that the asymmetry of MEX-5/6 is critical to maintain the pPAR domain. To test this, we set $D_f=D_s$ and controlled MEX-5/6 to be spatially homogeneous (Fig. 4d, left panels). The result showed that pPAR fails to maintain polarity and the polarity domain spread throughout the whole cell membrane. On the other hand, we found that the location of MEX-5/6 polarity does not affect pPAR polarity (Fig. 4d, right panels). To see how the location of MEX-5/6 polarity affects pPAR polarity, we switched the conversion roles of MEX-5/6 artificially in the MEX-5/6 model (11) such that

$$\begin{aligned} \begin{aligned}&\frac{\partial [M_{s}]}{\partial t}=D_{s}\nabla ^{2}[M_{s}] -\mu _4 \delta _3[M_{s}]+\mu _3 [P_c][M_{f}],&\text {on~}&{\mathbf {x}}\in \Omega , \\&D_{s}\frac{\partial [M_{s}]}{\partial {\mathbf {n}}} =-\mu _2 \frac{\delta _2}{(1+\mu _0[M])(1+\delta _1[P_m]^2)}[M_s]+\mu _1 [P_m][M_f],&\text {on~}&{\mathbf {x}} \in \partial \Omega ,\\&\frac{\partial [M_{f}]}{\partial t}=D_{f}\nabla ^{2}[M_{f}]-\mu _3 [P_c][M_{f}]+\mu _4 \delta _3[M_s],&\text {on~}&{\mathbf {x}}\in \Omega , \\&D_{f}\frac{\partial [M_{f}]}{\partial {\mathbf {n}}} =-\mu _1 [P_m][M_f]+\mu _2 \frac{\delta _2}{(1+\mu _0[M])(1+\delta _1[P_m]^2)}[M_s],&\text {on~}&{\mathbf {x}} \in \partial \Omega .\\ \end{aligned} \end{aligned}$$

Using this model, we controlled the location of MEX-5/6 polarity so that it had a high concentration in the posterior side. Unexpectedly, the simulation result showed that pPAR polarity is maintained robustly, even though the MEX-5/6 polarity is formed on the opposite site to the wild-type case. Taking these results together, we conclude that the location of MEX-5/6 polarity is not essential, but the asymmetry of MEX-5/6 distribution is indispensable for PAR polarity maintenance.

**Fig. 5:**
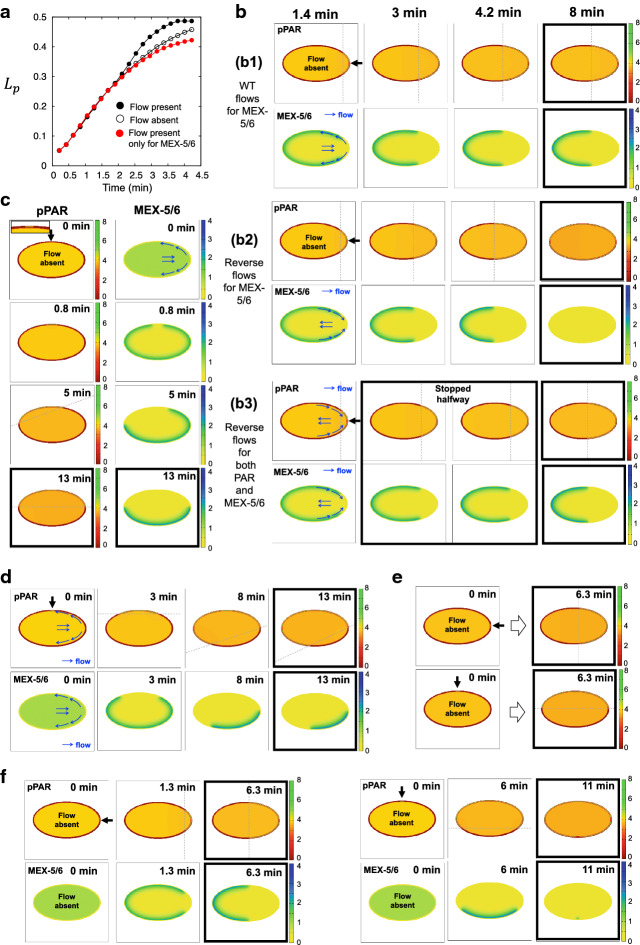
Interplay with the flows and cell geometry. **a** The effect of flow on the emerging speed. **b** The effect of flow direction on polarity patterning. The reverse flows are given by replacing $c_3$ and $c_8$ by $-c_3$ and $-c_8$, respectively, in the flow functions (12). **c** The effect of a different location of the symmetry breaking point without flow effects on pPAR dynamics. **d** The effect of a different location of the symmetry breaking point when wild-type flows are included. **e** pPAR polarity dynamics with a different location of symmetry breaking point when MEX-5/6 does not affect pPAR ($\mu _0=0$ in the model (10)–(11)) and flows are absent. **f** Comparison of polarity dynamics with different symmetry breaking points without flow effects. The figures surrounded by a bold square are the steady-state patterns. The gray dotted line indicates the boundary location of polarity domains. The detailed parameter values are given in “Appendix B”

### Interplay with the Flows and Cell Geometry

Cortical and cytoplasmic flows induced by acto-myosin contraction have been considered as critical factors in the patterning phase of PAR polarity formation (Goehring et al. 2011b). Nevertheless, it has not yet been explored how these flows can affect cytoplasmic polarity. To see how the cytoplasmic protein MEX-5/6 interacts with the flow dynamics and, consequently, affects the polarity dynamics, we have explored three cases: wild-type case, flow absent case, and flow present case only for MEX-5/6, in which the advection terms in the pPAR model (10) are removed. We first compared the temporal dynamics of polarity pattern for the aforementioned three cases (Fig. 5A). We found that the flows around the membrane can speed up the patterning time of pPAR, although the cytoplasmic flow in the bulk cytosol space has the opposite direction to that of the cortical flow (black dots and white dots in Fig. 5A). This indicates that the temporal dynamics of pPAR are affected strongly by the cortical flow, rather than the cytoplasmic flow.

On the other hand, we found that the interplay of MEX-5/6 and flows can slow down pattern emergence and negatively affect the temporal dynamics of patterning (red dots and white dots in Fig. 5A). This supposes that the cytoplasmic flows around the membrane transport the slow diffusive type of MEX-5/6 from the posterior pole to the anterior pole, resulting in lower MEX-5/6 concentration, and a weakening of the positive effect of MEX-5/6 for the pPAR to stay on the membrane (namely, either the inhibition effect on aPAR recruitment, or the activation effect on aPAR transmembrane off-rate). This result indicates that the flow dynamics do not always play a role in promoting PAR polarity, but can affect it negatively via the interplay with MEX-5/6. Nevertheless, such a negative effect is likely to be eliminated by the positive effect of the cortical flow on pPAR.

Next, we explored how the directions of flow interplay with MEX-5/6 dynamics and, consequently, influence the dynamics of PAR polarity. For this, we artificially imposed an opposite direction for the flows to the wild type only in MEX-5/6, with no flow in pPAR (Fig. 5B(b1),(b2)). We found that the flow directions greatly affected the dynamics of MEX-5/6, leading to completely different PAR polarity patterns. In contrast, there was no difference in the final polarity pattern (stationary steady state) in the case that both pPAR and MEX-5/6 are simultaneously affected by oppositely directed flows, although the temporal dynamics of patterning was affected when the velocity of flows was increased (Fig. 5B(b3)). This result indicates that when the flows affect pPAR and MEX-5/6 simultaneously, the influence of flow direction on the spatial dynamics of polarity can be negligible. To confirm this, we investigated the effect of the spatial position of symmetry breaking (Fig. 5C). We set the symmetry breaking position of pPAR polarity to be in a perpendicular location to that for the wild-type case but with the wild-type flow dynamics. This setting gives us the situation in which both pPAR and MEX-5/6 are strongly perturbed by flow. Nevertheless, we found that the final polarity domain is formed robustly, in the middle portion of the cell, even though the patterning phase is strongly affected by the flow directions (Fig. 5C). This result suggests that the MEX-5/6 is affected by the direction of flows, but its influence on spatial patterning can be greatly restricted by interaction with the PAR dynamics.

On the other hand, we found a very intriguing result by comparing Fig. 5B(b1) and D. In Fig. 5D, we considered only the flow effect for MEX-5/6 to be the same as Fig. 5B(b1) but with a different symmetry breaking position. That is, in these two numerical experiments, MEX-5/6 and pPAR are undergoing the same biochemical interactions under the same effect of cytoplasmic flows. However, the final polarity patterns are very different. Thus, we hypothesize that cell geometry may affect the state of biochemical interaction of cytoplasmic proteins and, consequently, results in different patterns. To confirm this, we investigated the effect of different symmetric breaking positions without flows (Fig. 5E, F). We found that this cell geometry effect is not seen in the PAR alone model, namely when we removed the effect of MEX-5/6 with $\mu _0=0$ (Fig. 5E), but the polarity pattern dynamics were dramatically changed, both temporally and spatially with respect to the cell geometry, when the effect of MEX-5/6 was included (Fig. 5F). This result proposes that the dynamics of cytoplasmic protein can be strongly affected by cell geometry and, consequently, the interplay between MEX-5/6 and cell geometry can lead to a dramatical change in PAR polarity dynamics. Summarizing the results, the effect of the interplay of MEX-5/6 with flows on the spatial dynamics of PAR polarity may be negligible, but the effect of cell geometry can be critical.

## Discussion

For the last ten years, the polarity phenomenon in asymmetric cell division has been extensively studied by both experimental and theoretical approaches. In particular, PAR polarity in the membrane has been found to be the most upstream regulator which controls the dynamics of cytoplasmic polarity. However, how downstream polarity in the cytosol affects PAR polarity, and how they interact, is still unknown. In this study, we revisited the question of polarity formation of the cytoplasmic protein MEX-5/6 by combining the dynamics of the upstream protein PAR and explored how the cytoplasmic polarity of MEX-5/6 interacts with the membrane PAR polarity in the high-dimensional bulk-surface model.

The mechanism for generating MEX-5/6 polarity has been studied in MEX-5/6 alone models (Daniels et al. 2010; Wu et al. 2018), which assumed that the conversion rate of MEX-5/6 diffusion is spatially heterogeneous, and did not include PAR dynamics. By contrast, we have developed a conversion model in which the conversion rate functions of the two diffusion types of MEX-5/6 are determined by PAR proteins in a concentration-dependent manner, so that the model formulation directly includes the dynamics of PAR proteins. In our model, we supposed that the conversion rate function from fast type to slow type depends on the concentration of aPAR. However, this term was converted to a suppression term by pPAR concentration in the self-recruitment model (Sect. 2.4). The formulation of our self-recruitment model implicitly includes the dynamics of PAR-1, which acts repressively on the phosphorylated substance PP2A, converting the fast type of MEX-5/6 to the slow type (Griffin et al. 2011). In fact, for the conversion dynamics of MEX-5/6 diffusion type, Daniels et al. (2010) assumed a direct conversion from fast type to slow type by aPAR. In contrast, Griffin et al. (2011) proposed that pPAR represses the conversion of MEX-5/6 from fast diffusive type to the slow type. However, our model formulation and analysis showed that such apparently contrasting propositions are essentially the same. This suggests that the conversion assumptions of our model capture an essential mechanism of interplay between cytoplasmic protein MEX-5/6 and PAR proteins, which may suggest a general mathematical structure for pattern formation in cytoplasmic proteins.

With our MEX-5/6-combined-pPAR model, we explored the specific role of MEX-5/6 on PAR polarity formation. We found that MEX-5/6 can play a critical role in inducing the symmetry breaking. Motegi et al. (2011) showed that the recruitment of PAR-2 around the posterior pole by microtubule transport may promote symmetry breaking. Our study suggests that symmetry breaking can also be promoted by the repression of aPAR recruitment around the posterior pole by MEX-5/6, implying that the self-recruitment of pPAR is indirectly promoted. These results propose that the first stage of symmetry breaking in asymmetric division may be regulated by the synergistic effect of multiple positive feedbacks of pPAR recruitment from cytosol to membrane. We also found that the length of the pPAR domain tends to be shorter as the regulation effect ($\mu _0$) of MEX-5/6 on PAR decreases. This is consistent with the dynamics observed in previous experiments with the *mex-5(RNAi)* and *mex-6(RNAi)* embryos of *C. elegans*, where the length of the PAR-2 domain was shorter than that of the wild type (Cuenca et al. 2002; Schubert et al. 2000). However, we also found that the length scale of the pPAR polarity domain is not sensitive to the regulation effect of MEX-5/6, indicating that the length of the PAR domain may be robustly regulated by other factors, such as the total mass of PAR proteins (Seirin-Lee and Shibata 2015; Goehring et al. 2011b). Our study suggests that the upstream polarity of the PARs, and the downstream polarity of MEX-5/6, significantly regulate each other with respect to both spatial and temporal dynamics in polarity formation. Even if cytoplasmic polarity serves as a downstream regulator, cytoplasmic polarity can play a critical role in upstream PAR polarity, and the balance of their bi-directional regulation is important for generating robust polarity.

In the history of the study of pattern formation, the effect of domain geometry has been considered as an important factor that can regulate spatial patterning (Crampin et al. 1999; Dawes and Iron 2013; Murray 1993; Seirin-Lee 2017), and there is biological evidence supporting the hypothesis that the shape, or size, of domain is likely to play a critical role in determining cell function, via regulation of pattern formation (Kondo and Asai 1995; Seirin-Lee et al. 2019). In this study, we found that cell geometry may play an important role in the dynamics of cytoplasmic protein in polarity formation. Many cell polarity studies using mathematical models have been focused on the dynamics of membrane polarity in simplified one-dimensional domains, neglecting cell geometry. In general, the fast diffusion in cytosol, and the homogeneity of cytosol concentration, have validated this model simplification. However, our study suggests that the effect of cell geometry on the cytoplasmic protein, which creates a spatial heterogeneity in the bulk cytosol space, can play a critical role in the dynamics of polarity patterning in both membrane and cytosol, and that cell geometry should not be neglected. Furthermore, the flow dynamics is likely to be affected by cell geometry (Mittasch et al. 2018), which may consequently affect PAR polarity formation.

In this study, we presented simulation results for representative parameter sets. However, a rigorous mathematical analysis of the high-dimensional bulk-surface model of PARs alone proves that the polarity pattern exists within a large parameter range (Morita and Seirin-Lee 2020). Furthermore, our bi-stability analysis shows that the parameter region can be extended as the effect of MEX-5/6 increases (Fig. S2B), implying that we could have a polarity pattern in the MEX-5/6-combined-PAR model which is robust to the values of the kinetic parameters. However, it is a mathematical challenge to analyze the bifurcation structure, existence of the polarity solution, and the details of polarity dynamics, in the high-dimensional bulk-surface MEX-5/6-combined-PARs system.

Finally, our study proposes that to understand the whole process of cell polarity in asymmetric cell division, it is vital to integrate biochemical interaction, biophysical dynamics, and cell geometry.

### Supplementary Information

Below is the link to the electronic supplementary material.

Supplementary material 1 (mov 302 KB)

Supplementary material 2 (mov 80 KB)

Supplementary material 3 (mov 74 KB)

Supplementary material 4 (pdf 200 KB)

Supplementary material 5 (pdf 34 KB)

## Sharp Interface Limit and Zero-Flux Boundary Condition

We show that a sharp interface limit recovers the boundary conditions in the cytosol equations of the phase-field combined model (14). The general form of the cytoplasmic protein dynamics in our model is given by

15 $$\begin{aligned} \frac{\partial u}{\partial t}+\nabla \cdot ({\mathbf {v}}u)&=D\nabla ^2 u \text { on } {\mathbf {x}}\in \Omega , \end{aligned}$$

16 $$\begin{aligned} D\frac{\partial u}{\partial {\mathbf {n}}}-{\mathbf {v}}u&=-F(u) \text { on } {\mathbf {x}}\in \Gamma , \end{aligned}$$

where *u* is the concentration of cytoplasmic protein, *D* is the diffusion coefficient, ${\mathbf {v}}$ is the flow velocity, and ${\mathbf {n}}$ is the normal vector on $\Gamma (\equiv \partial \Omega )$. *F*(*u*) is a function describing a reaction on $\Gamma$ and it is not necessarily only a function of *u*. The cytoplasmic model equation incorporating cell shape is given by

17 $$\begin{aligned} \phi \frac{\partial u}{\partial t}+\nabla \cdot (\phi {\mathbf {v}}u)=D\nabla \cdot (\phi \nabla u)+\nabla \phi F(u) \text { on } {\mathbf {x}}\in {\mathbb {R}}^N, \end{aligned}$$

where $\phi$ is the phase-field function at a fixed time, *i.e.*, $\phi =\phi ({\mathbf {x}})$, defined by

$$\begin{aligned} \phi ({\mathbf {x}})= & {} 1 \text { on cytosol}, \quad 0<\phi ({\mathbf {x}})<1 \text { on cell membrane}, \quad \\ \phi ({\mathbf {x}})= & {} 0 \text { on extracellular space}. \end{aligned}$$

In what follows, we show that Eq. (17) recovers the boundary condition (16) in the sharp interface. Let us define $T_\xi$ to be the interface region of $\phi$ with thickness $\xi$. That is,

$$\begin{aligned} T_\xi =\{{\mathbf {x}}\in {\mathbb {R}}^{N}| 0<\phi ({\mathbf {x}})<1 \} \quad \text {and} \quad \lim _{\xi \rightarrow 0}T_{\xi }=\Gamma . \end{aligned}$$

Integrating Eq. (17) over the interface yields

18 $$\begin{aligned} \begin{aligned} \int _{T_\xi }\phi \frac{\partial u}{\partial t}\ \mathrm{d}V&= \int _{T_\xi }\nabla \cdot (D\phi \nabla u-\phi {\mathbf {v}}u)\ \mathrm{d}V+\int _{T_\xi }\nabla \phi F(u)\ \mathrm{d}V,\\&=\int _{\partial T_{\xi }}(D\phi \nabla u-\phi {\mathbf {v}}u)\cdot {\mathbf {n}}\ \mathrm{d}S+\int _{T_\xi }\nabla \phi F(u)\ \mathrm{d}V,\\&=\int _{\partial T_{\xi }}\phi (D\nabla u-{\mathbf {v}}u)\cdot {\mathbf {n}}\ \mathrm{d}S+\int _{T_\xi }\nabla \phi F(u)\ \mathrm{d}V. \end{aligned} \end{aligned}$$

On the other hand, substituting the original Eq. (15) into the left-hand side of equation (18) gives

19 $$\begin{aligned} \begin{aligned} \int _{T_\xi }\phi \frac{\partial u}{\partial t}\ \mathrm{d}V&=\int _{T_\xi }\phi \nabla \cdot (D\nabla u-{\mathbf {v}}u)\ \mathrm{d}V\\&=\int _{\partial T_\xi }\phi (D\nabla u -{\mathbf {v}}u)\cdot {\mathbf {n}}\ \mathrm{d}S-\int _{T_\xi }\nabla \phi \cdot (D\nabla u - {\mathbf {v}}u)\ \mathrm{d}V. \end{aligned} \end{aligned}$$

Thus, from (18) and (19), we obtain

$$\begin{aligned} \int _{T_\xi }\nabla \phi \cdot \left[ D\nabla u - {\mathbf {v}}u+F(u)\right] \ \mathrm{d}V=0. \end{aligned}$$

Since $$\nabla \phi \ne 0$$ as $$\xi \rightarrow 0$$,

$$\begin{aligned} -D\frac{\partial u}{\partial {\mathbf {n}}}+{\mathbf {v}}u=F(u) \quad \text { as } \xi \rightarrow 0. \end{aligned}$$

should hold.

## Initial Conditions and Parameter Values

Before fertilization, aPAR is homogeneously distributed in the membrane, and pPAR and MEX-5/6 are homogeneously distributed in the cytosol. The core mechanism for inducing symmetry breaking remains unclear, as does the reason why the pPAR domain begins around the posterior polar site. On the other hand, it is known that some signals from the centrosome and its microtubule asters help recruit pPAR into the membrane around the posterior polar site (Rose and Gönczy 2014; Motegi et al. 2011; Motegi and Seydoux 2013). Thus, we define the initial conditions such that all proteins are at homogeneous steady state, and some small stimulus of pPAR is added in a small region of the membrane.

The detailed form of the initial conditions are :

$$\begin{aligned}&[A_{m}]({\mathbf {X}},0)c[A_{m}]_{0}(1+\delta \psi ({\mathbf {X}}))\, \text {on} \,\ {\mathbf {X}}\in \partial \Omega , \\&[A_{c}]({\mathbf {X}},0)=[A_{c}]_{0}(1+\delta \psi ({\mathbf {X}})) \,\text {on} \,\ {\mathbf {X}}\in \Omega , \\&[P_{m}]({\mathbf {X}},0)=[P_{m}]_{0}+\sigma \,\text {on} \,\ {\mathbf {X}}\in \omega \subset \partial \Omega , \\&[P_{m}]({\mathbf {X}},0)=[P_{m}]_{0}(1+\delta \psi ({\mathbf {X}}))\,\text {on}\, \ {\mathbf {X}}\in \partial \Omega \setminus \omega , \\&[P_{c}]({\mathbf {X}},0)=[P_{c}]_{0}(1+\delta \psi ({\mathbf {X}})) \,\text {on}\, \ {\mathbf {X}}\in \Omega , \\&[M_{f}]({\mathbf {X}},0)=[M_{f}]_{0}(1+\delta \psi ({\mathbf {X}})) \,\text {on}\, \ {\mathbf {X}}\in \Omega , \\&[M_{s}]({\mathbf {X}},0)=[M_{s}]_{0}(1+\delta \psi ({\mathbf {X}}))\,\text {on}\, \ {\mathbf {X}}\in \Omega , \end{aligned}$$

where $\sigma$ is the strength of the signal, and $\omega$ is the sufficiently small region in which the signal is imposed. $\delta$ is a positive constant and has a very small value. Typically, we set $\delta =0.02$. $\psi ({\mathbf {X}})$ is a random function with uniform distribution, which takes values in the range $[-0.5,0.5]$. $[A_{m}]_{0}$, $[A_{c}]_{0}$, $[P_{m}]_{0}$, $[P_{c}]_{0}$, $[M_{f}]_{0}$ and $[M_{s}]_{0}$ are equilibrium concentrations. We set $[A_{m}]_{0}, [P_{m}]_0$, $[M_s]_{0}$ and $[M_f]_{0}$, directly, where $[A_{c}]_{0}$ and $[P_{c}]_{0}$ are calculated as the equilibrium values in model (1)–(3), or (10)–(11) when $\mu _0=0$.

**Table 1:** Representative parameter set. ${\mathcal {M}}$ has the scale of protein number used to non-dimensionalize the model

Parameter	Dimensional value	Dimensionless value
$^{ \dagger }\ell _x$	30.0 ($\upmu$m)	0.4
$^{ \dagger }\ell _y$	16.875 ($\upmu$m)	0.225
*t*	0.3375 (*s*)	1.0
$^{ \dagger }D^{P}_{m}$	0.120 ($\upmu \text {m}^{2}/\mathrm{s}$)	7.2$\times 10^{-6}$
$^{ \ddagger }D^{P}_{c}$	6.0 ($\upmu \text {m}^{2}/\mathrm{s}$)	3.6$\times 10^{-4}$
$^{ *}D_{f}$	8.5 ($\upmu \text {m}^{2}/\mathrm{s}$)	5.134$\times 10^{-4}$
$^{ *}D_{s}$	0.59 ($\upmu \text {m}^{2}/\mathrm{s}$)	3.54$\times 10^{-5}$
$\gamma _{2}$	5.9 ($\mathrm{s}^{-1}$)	2.0
$\alpha _{2}$	1.8 ($\mathrm{s}^{-1}$)	0.6
$\beta _{1}$	0.59 ($\mathrm{s}^{-1}$)	0.2
$\beta _{2}$	0.15 ($\mathrm{s}^{-1}$)	0.05
$\beta _{3}$	2.07 ($\mathrm{s}^{-1}{\mathcal {M}}^{-2}$)	0.7
$\beta _{4}$	100.93 ($\mathrm{s}^{-2}$)	12.45
$\mu _{0}$	$0.1~({\mathcal {M}}^{-1})$	0.1
$\mu _{1}$	0.18 ($\mathrm{s}^{-1}$)	0.05
$\mu _{2}$	0.59 ($\mathrm{s}^{-1}$)	0.2
$\mu _{3}$	0.015 ($\mathrm{s}^{-1}$)	0.005
$\mu _{4}$	0.015 ($\mathrm{s}^{-1}$)	0.005
$\delta _{1}$	4.5 (${\mathcal {M}}^{-2}$)	4.5
$\delta _{2}$	5.0 (${\mathcal {M}}$)	5.0
$\delta _{3}$	0.5 (${\mathcal {M}}$)	0.5

$^{\dagger }$Goehring et al. (2011a),$^{\ddagger }$Kuhn et al. (2011), $^{*}$Wu et al. (2018)

We selected the parameter values based on experimental data for the *C. elegans* embryo. A fertilized *C. elegans* egg is usually elliptical, and the radii of the long and short axes are observed typically in the range of $27.0\pm 1.7 ~\upmu \mathrm{m}$ and $14.8\pm 1.0 ~\upmu \mathrm{m}$, respectively (Goehring et al. 2011a). The diffusion rates of aPAR and pPAR in the membrane were selected based on the diffusion rate of PAR-6 and PAR-2, respectively (Goehring et al. 2011a). The cytoplasmic diffusion rates of aPAR and pPAR were chosen from the data in Kuhn et al. (2011). For the diffusion rates of MEX-5/6, we used data from Daniels et al. (2010) and Wu et al. (2018), and for the flow velocity, we used data from Goehring et al. (2011a) and Niwayama et al. (2011). We selected the time scale to coincide with the quantitative data and the qualitative dynamics of PAR polarity. The unit time in the non-dimensionalized system corresponds to the dimensional time, 0.3375 s. In the simulations, we used the non-dimensionalized model, combined with the phase-field functions, and solved the equations on the non-dimensional region $1\times 1$, where the dimensional space $L_x\times L_y$ has been scaled by $L_x=L_y=75~\upmu \mathrm{m}$ (Fig. 1a). Since the phase-field function is time-independent and is used to define the cell region, we assume the parameters used in the phase-field function as dimensionless quantities. The parameter values are listed in Table 1, and the detailed parameter values used in the flow functions, phase-field function, and the figures are given in the following. For the other figures, the parameter values in Table 1 were used.

- Flow functions, (12): $$ c_1 = 0.001, ~c_2 = 400.0, ~c_3 = 0.02, ~c_4 = 4.2, ~c_5 = 0.01,~c_6 = 0.00001,~c_7 = 3.5,~c_8 = 0.0021,~c_9 = 0.00005, ~T_0 = 3.3375 ~\text {min}$$
- Phase-field function, (13): $\alpha =120,~\overline{V}=0.2828, \mu =1.0,~\varepsilon =2.0\times 10^{-3}, ~\nu =16.0$
- Figure 1a: $$D^{A}_{m}=0.000001652, ~D^{A}_{c}=0.00036, ~D^{P}_{m}=0.00000072,~D^{P}_{c}=0.00036, \gamma _1=\gamma _2=0.3, ~\alpha _1=\alpha _2=0.06,~K_1=K_3=0.4, ~K_2=K_4=1.0,~\overline{K}_2=\overline{K}_4=0.05,~\mu _0=0.01,~\mu _1=0.05,~\mu _2=0.01,~\mu _3=\mu _4=0.005$$.
- Figure 4a: $\beta _3=0.5$, $\mu _0=0$ or 0.2, and the other parameters are given in Table 1.
- Figure 4b: $\mu _0=0.2$ and the other parameters are given in Table 1.

## Incompressibility

The detailed figure of incompressibility is shown in Fig. S1.

## Supporting Role of MEX-5/6 on pPAR Patterning

Here, we show that MEX-5/6 can play a supporting role in inducing pPAR polarity patterning by analyzing a parameter space where bi-stability is possible. Let us assume that the concentration of MEX-5/6 is given by a constant, namely, $M^*$. Then, from Eq. (10), the equilibrium state of pPAR can be written as

20 $$\begin{aligned} \gamma _2 P_{c}^*-\left\{ \alpha _2+\frac{\beta _4}{\beta _1+(1+\mu _0 M^*)^2(\beta _2+\beta _3 (P_m^*)^2)} \right\} P_m^*=0, \end{aligned}$$

where $P_{c}^*$ and $P_m^*$ are the equilibrium concentrations of pPAR in the cytosol and the membrane, respectively. Note that

21 $$\begin{aligned} P_{c}^*=\frac{1}{|\Omega |}\int _{\Omega }P_{c}^*=\frac{1}{|\Omega |}\left( P_\mathrm{tot}-\int _{\partial \Omega }P_{m}^*\right) =P_\mathrm{tot}^*-AP_{m}^*, \end{aligned}$$

where $P_\mathrm{tot}=\int _{\Omega }P_{c}+\int _{\partial \Omega }P_{m}$, $P_\mathrm{tot}^*=P_\mathrm{tot}/|\Omega |$, and $A=|\partial \Omega |/|\Omega |$. Substituting Eq. (21) into Eq. (20), we obtain

$$\begin{aligned} G(P_m^*)&=-(\alpha _2+A\gamma _2)(P_m^*)^3+P_\mathrm{tot}^*\gamma _2(P_m^*)^2\\&\quad -\frac{(\alpha _2+A\gamma _2)(\beta _1+\beta _2(1+\mu _0M^*)^2+\beta _4)}{\beta _3(1+\mu _0M^*)^2}P_m^* \\&\quad +\gamma _2P_\mathrm{tot}^*\frac{\beta _1+\beta _2(1+\mu _0M^*)^2}{\beta _3(1+\mu _0M^*)^2}=0. \end{aligned}$$

For two positive stable states, the equation $G'(p_m^*)=0$ should have two positive solutions (Fig. S2A). Denoting the two solutions by $P_1$ and $P_2$, the following property is satisfied :

22 $$\begin{aligned} P_1+P_2>0, \qquad P_1P_2>0, \end{aligned}$$

and

$$\begin{aligned} \beta _4<\left[ \frac{\beta _3(P_\mathrm{tot}^*\gamma _2)^2}{3(\alpha _2+A\gamma _2)}-(\alpha _2+A\gamma _2)\beta _2 \right] (1+\mu _0M)^2-(\alpha _2+A\gamma _2)\beta _1. \end{aligned}$$

We can easily check that the conditions (22) are satisfied for all positive parameter values. Thus, we compare the parameter region of $\gamma _2$ (on-rate) and $\beta _4$ (magnitude of off-rate) for bi-stability with respect to the cases $\mu _0=0$ and $\mu _0=0.2>0$. The result shows that the case of $\mu _0>0$ gives a wider parameter space for bi-stability than the case of $\mu _0=0$.
