# Supplementary figures and images for: The Role of Cytoplasmic MEX-5/6 Polarity in Asymmetric Cell Division
Source: Bull Math Biol. 2021 Feb 17;83(4):29. doi: 10.1007/s11538-021-00860-0 (PMC7886744; doi:10.1007/s11538-021-00860-0)

## $\nabla \cdot v$ in the cytosol

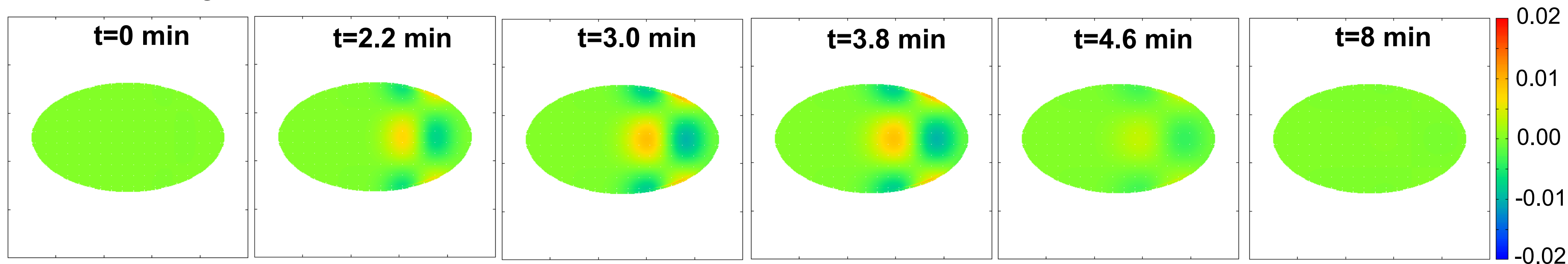

## $\nabla \cdot v$ in the membrane

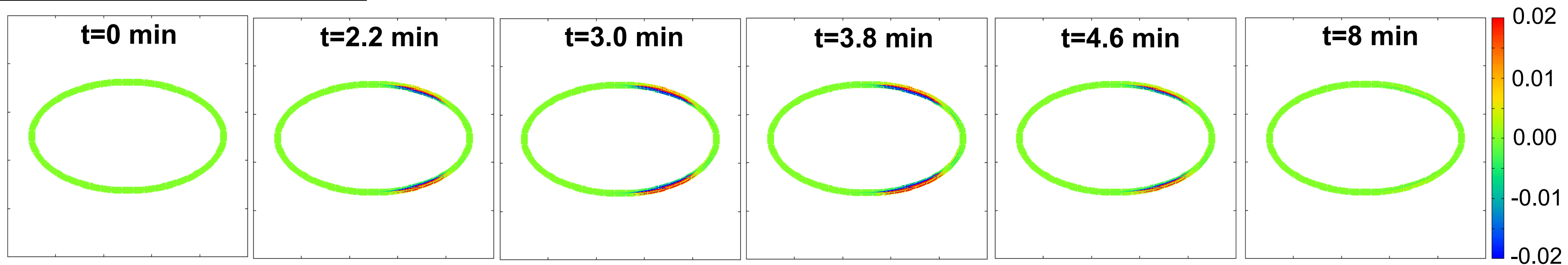

Supplement: Supplementary file 4 — Supplementary material 4 (pdf 200 KB) [file 11538_2021_860_MOESM4_ESM.pdf]

**A**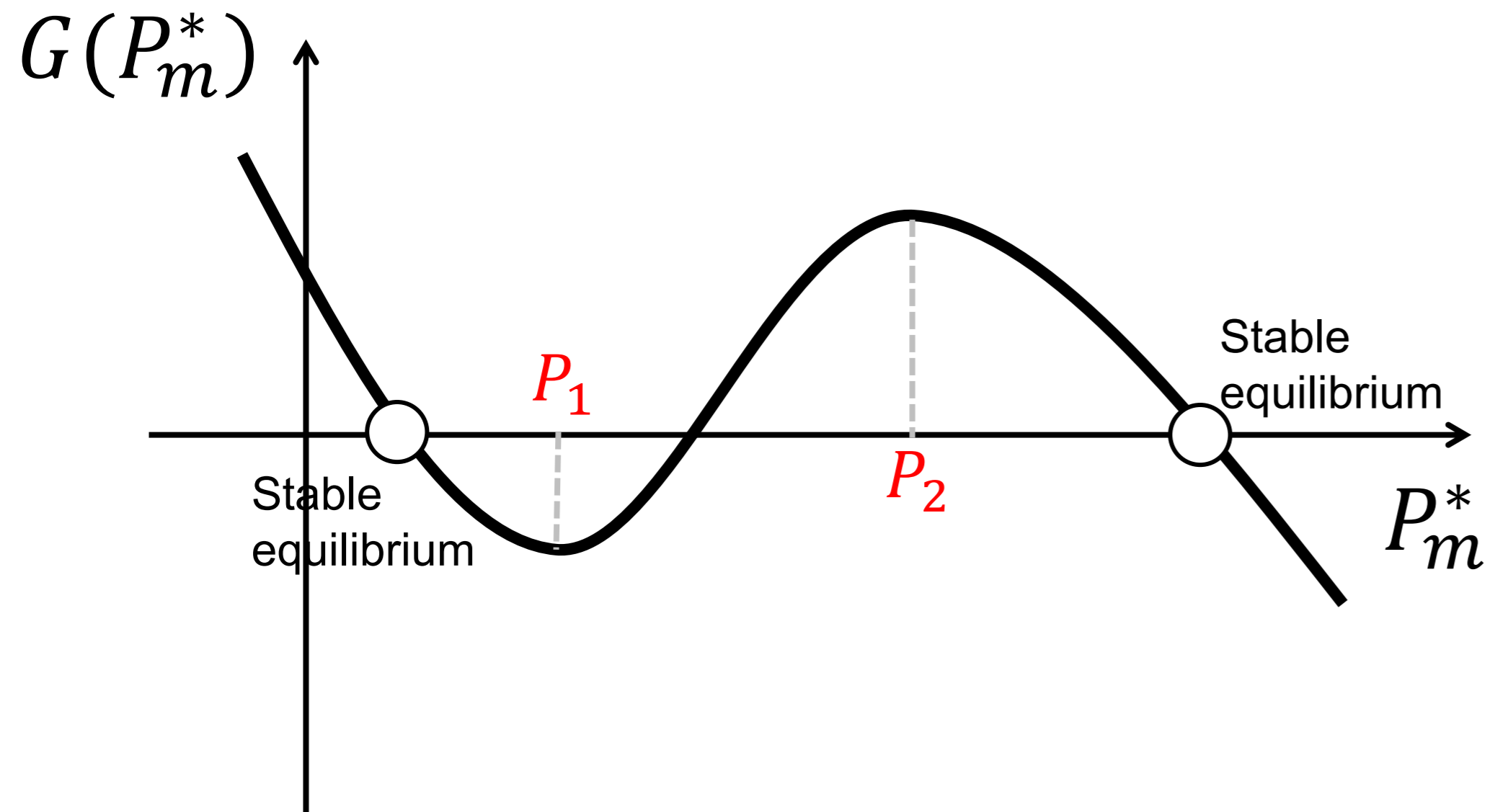**B**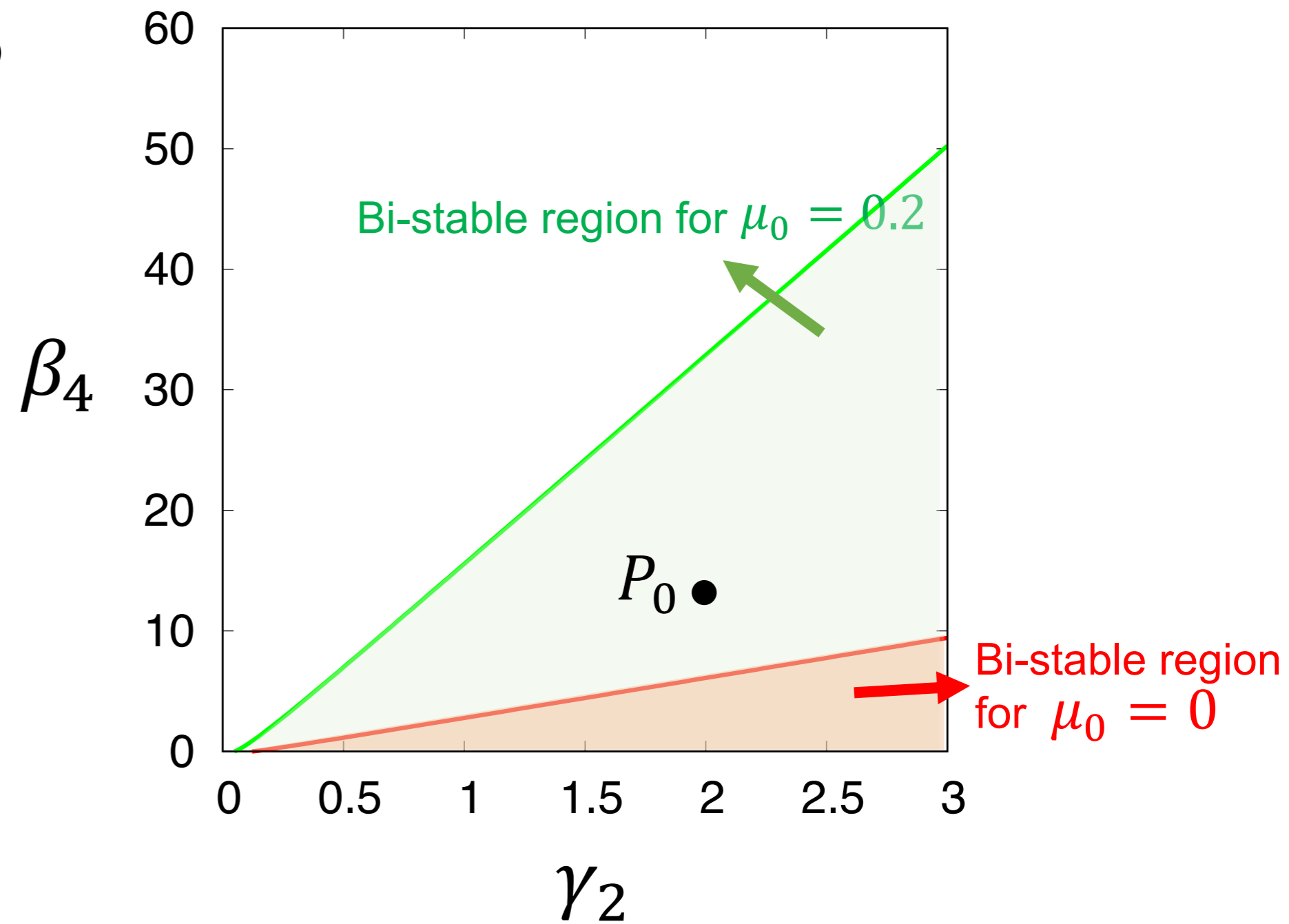

Supplement: Supplementary file 5 — Supplementary material 5 (pdf 34 KB) [file 11538_2021_860_MOESM5_ESM.pdf]
